# Orai3 Calcium Channel Contributes to Oral/Oropharyngeal Cancer Stemness through the Elevation of ID1 Expression

**DOI:** 10.3390/cells12182225

**Published:** 2023-09-07

**Authors:** Anthony Nguyen, Youngjae Sung, Sung Hee Lee, Charlotte Ellen Martin, Sonal Srikanth, Wei Chen, Mo K. Kang, Reuben H. Kim, No-Hee Park, Yousang Gwack, Yong Kim, Ki-Hyuk Shin

**Affiliations:** 1 The Shapiro Family Laboratory of Viral Oncology and Aging Research, UCLA School of Dentistry, Los Angeles, CA 90095, USA; nguyenanthony45@ucla.edu (A.N.);; 2 Department of Physiology, David Geffen School of Medicine at UCLA, Los Angeles, CA 90095, USA; 3 UCLA Jonsson Comprehensive Cancer Center, Los Angeles, CA 90095, USA; 4 Department of Medicine, David Geffen School of Medicine at UCLA, Los Angeles, CA 90095, USA; 5 Laboratory of Stem Cell and Cancer Epigenetics, UCLA School of Dentistry, Los Angeles, CA 90095, USA; 6 UCLA Broad Stem Cell Research Center, Los Angeles, CA 90095, USA

**Keywords:** calcium, Orai3, OSCC, cancer stem-like cells, ID1

## Abstract

Emerging evidence indicates that intracellular calcium (Ca^2+^) levels and their regulatory proteins play essential roles in normal stem cell proliferation and differentiation. Cancer stem-like cells (CSCs) are subpopulations of cancer cells that retain characteristics similar to stem cells and play an essential role in cancer progression. Recent studies have reported that the Orai3 calcium channel plays an oncogenic role in human cancer. However, its role in CSCs remains underexplored. In this study, we explored the effects of Orai3 in the progression and stemness of oral/oropharyngeal squamous cell carcinoma (OSCC). During the course of OSCC progression, the expression of Orai3 exhibited a stepwise augmentation. Notably, Orai3 was highly enriched in CSC populations of OSCC. Ectopic Orai3 expression in non-tumorigenic immortalized oral epithelial cells increased the intracellular Ca^2+^ levels, acquiring malignant growth and CSC properties. Conversely, silencing of the endogenous Orai3 in OSCC cells suppressed the CSC phenotype, indicating a pivotal role of Orai3 in CSC regulation. Moreover, Orai3 markedly increased the expression of inhibitor of DNA binding 1 (ID1), a stemness transcription factor. Orai3 and ID1 exhibited elevated expression within CSCs compared to their non-CSC counterparts, implying the functional importance of the Orai3/ID1 axis in CSC regulation. Furthermore, suppression of ID1 abrogated the CSC phenotype in the cell with ectopic Orai3 overexpression and OSCC. Our study reveals that Orai3 is a novel functional CSC regulator in OSCC and further suggests that Orai3 plays an oncogenic role in OSCC by promoting cancer stemness via ID1 upregulation.

## 1. Introduction

Recent studies have uncovered and validated the pathophysiological significance of cancer stemness, referring to the stem cell-like phenotype of cancers, in the long-term sustenance of cancers [1]. Cancer stemness is known to be responsible for tumorigenicity, metastasis, resistance to therapy, and the recurrence of cancer cells, indicating its pivotal role in tumor progression and aggression [2]. The stemness phenotype has been observed in various human cancers, including OSCC [2]. OSCC, the sixth most prevalent cancer worldwide, often arises subsequent to clinically well-defined lesions, notably leukoplakia, which is histologically categorized as either dysplastic or non-dysplastic leukoplakia. Dysplastic leukoplakia is characterized as an oral premalignant lesion and is linked to a probable progression to cancer. However, it does not reliably predict the risk of cancer [3,4]. Early-stage tumors can typically be addressed through surgical procedures and radiotherapy. Nonetheless, the efficacy of treatment is inversely proportional to the extent of the disease progression at the time of intervention. While a combination of chemotherapy and radiation therapy proves effective in treating 97% of early-stage tumors, its effectiveness dwindles to 33% for advanced tumors [5]. Hence, advancing our understanding of the molecular mechanisms governing cancer stemness is crucial for developing a new generation of effective therapies for OSCC.

Intracellular Ca^2+^ is a universal second messenger regulating many physiological processes; the deregulation of intracellular Ca^2+^ is implicated in cell proliferation, metastasis, and suppression of apoptosis, indicating that altered Ca^2+^ homeostasis is a hallmark of cancer cells [6,7,8,9]. Emerging evidence also indicates the crucial role of Orai Ca^2+^ channels in carcinogenesis [10,11,12,13,14,15,16,17,18,19,20,21,22,23,24,25,26,27,28]. The Orai channel family comprises three Orai homologs (Orai1, 2, and 3), distinguished by their pronounced calcium selectivity. These channels mediate intracellular Ca^2+^ influx in non-excitable cells, such as cancer cells [29]. Among the three Orai homologs, Orai1, the most studied homolog, was reported to play a critical role in cancer progression [30]. We demonstrated that increased Orai1 expression is necessary and sufficient for tumor progression by promoting cancer stemness in OSCC [14]. Our study further showed that the activation of Ca^2+^-dependent transcription factor, NFAT (nuclear factor of activated T cells) was required for Orai1-induced stemness promotion in OSCC, indicating the novel role of the Orai1/NFAT axis in cancer stemness [14]. The oncogenic role of Orai2 has been demonstrated in various human cancers including OSCC [31]. Similar to Orai1, Orai2 was significantly upregulated in OSCC tissues in comparison to normal tissues [31]. The inhibition of Orai2 suppressed malignant growth and properties in OSCC cells [31]. These findings indicate that Orai1 and Orai2 have significant roles in OSCC; however, the role of Orai3 in OSCC remains poorly explored especially in cancer progression and stemness.

In this current research, we introduce a novel finding: the elevated expression of Orai3 in oral and oropharyngeal carcinogenesis occurs in a gradual fashion and is notably concentrated within OSCC CSC (cancer stem-like cell) populations. Additionally, we have furnished compelling evidence that Orai3 significantly advances the progression of OSCC by amplifying cancer stemness through the elevation of ID1 expression. This underscores an innovative regulatory mechanism for cancer stem cells involving the Orai3/ID1 axis.

## 2. Materials and Methods

### 2.1. Cell Culture and Reagents

Primary normal human oral keratinocytes (NHOK) were prepared from oral mucosa and cultured in Keratinocyte Growth Medium (KGM, Lonza, Los Angeles, CA, USA). Non-tumorigenic immortalized oral epithelial cell lines HOK-16B were also cultured in KGM. Eleven human OSCC cell lines (BapT, FaDu, SCC4, SCC9, SCC9/TNF, SCC15, SCC105, SNU1066, UM6, UM17, and YD38) were cultured in DMEM/Ham’s F12 (Invitrogen, Waltham, MA, USA) supplemented with 10% FBS (Gemini Bioproducts, West Sacramento, CA, USA), 0.4 μg/mL hydrocortisone (Sigma-Aldrich, St. Louis, MO, USA) and 5 μg/mL Gentamycin aminoglycoside antibiotic (Invitrogen).

### 2.2. Laser-Captured Microdissection (LCM)

Following histological examination of H & E staining of oral mucosal tissues (i.e., NHOE (*n* = 4), precancerous oral lesions (*n* = 4) and OSCC tissues (*n* = 4)), epithelial layers from the paraffin-embedded tissue samples were excised by laser-captured microdissection (LCM) using Leica (LMD) 7000 system (Leica Microsystems Inc., Richmond, IL, USA) at the California NanoSystems Institute at UCLA (Los Angeles, CA, USA). LCM-derived tissue RNAs were extracted using a high pure RNA paraffin kit (Roche, Basel, Switzerland). RT was performed with RNA isolated from the tissue sections using a Superscript II RT kit (Invitrogen) with random hexamer primers (Promega, Madison, WI, USA) according to the manufacturer’s instructions. Then, qPCR was performed using PowerUp SYBR Green Master Mix (Thermo Fisher Scientific, Waltham, MA, USA) and QuantStudio 3 qPCR System (Thermo Fisher Scientific) as described in our prior work [32]. Thermocycling conditions for all PCR reactions included an initial denaturation stage at 95 °C for 10 min, followed by 50 cycles.

### 2.3. Quantitative Real-Time PCR (qPCR)

A total of 2.5 μg of total RNA was utilized for the synthesis of cDNA using the SuperScript first-strand synthesis system from Invitrogen. Subsequently, quantitative PCR (qPCR) was conducted using the PowerUp SYBR Green Master Mix provided by Thermo Fisher Scientific, along with the QuantStudio 3 qPCR System, consistent with the procedures outlined in our prior publication [14]. Primer sequences were sourced from the Universal Probe Library by Roche, and these sequences can be made available upon request. The determination of fold-differences was performed using the second derivative Cq value method in accordance with the instructions provided by the manufacturer.

### 2.4. Western Blotting

Western blotting was performed as described previously [14]. We used the following primary antibodies: anti-Orai3 (ab115558, Abcam, Cambridge, UK), anti-ID1 (sc-133104, Santa Cruz, CA, USA), anti-ID2 (sc-398104, Santa Cruz, CA, USA), anti-Bmi1 (5856, Cell Signaling), anti-EZH2 (ab186006, Abcam, Cambridge, UK), anti-Nanog (sc-293121, Santa Cruz, CA, USA), anti-Oct4 (sc-5279, Santa Cruz, CA, USA), and anti-GAPDH (sc-25778; Santa Cruz, CA, USA). Horseradish peroxidase (HRP)-conjugated secondary antibodies were obtained from Santa Cruz.

### 2.5. Tumor Sphere Formation Assay

A total of three thousand cells were cultivated in 3 mL of serum-free DMEM/F12 media, which was supplemented with a 1:50 dilution of B27 from Invitrogen, along with 20 ng/mL of EGF, and 10 μg/mL of insulin. This culture medium was also augmented with penicillin, streptomycin, and amphotericin B. The cells were grown in ultra-low attachment 6-well plates provided by Corning, and this cultivation process took place over a span of 6–10 days. The resulting formation of tumor spheres was subsequently observed and quantified through microscopic examination.

### 2.6. Migration Assay

The measurement of cell migration was conducted using 6.5 mm transwell chambers featuring 8.0 μm polycarbonate membranes (Corning: Product#3422), in accordance with the methodology outlined in our earlier publication [14]. Specifically, a total of twenty thousand cells were seeded into the chambers and subsequently incubated for a duration of 2 days.

### 2.7. Ectopic Expression of Orai3

For the preparation of viruses, the retroviral pMSCV-CITE-eGFP-Puro vector containing human Orai3 [33] was employed. This vector was transfected into GP2-293 universal packaging cells (Clonetech, Mountain View, CA, USA) together with the pVSV-G envelope plasmid using the lipofectamine 2000 transfection reagent from Life Technologies. A comprehensive description of retrovirus production and infection methodologies can be located in our previous publications [14]. Following infection, the cells were subjected to a selection process using 0.5 μg/mL of puromycin over a span of two weeks. These selected cells were subsequently utilized for the experimental procedures.

### 2.8. SOCE Assay (Single-Cell Ca^2+^ Imaging)

The day before imaging, cells were seeded onto UV-sterilized coverslips. On the subsequent day, the cells were subjected to loading with 1 mM Fura 2-AM for a duration of 45 min at a temperature of 25 °C. Intracellular calcium ion concentration ([Ca^2+^]_i_) measurements were conducted using methods that were largely consistent with those previously described [14,34].

### 2.9. Cell Proliferation Assay

Cell growth assessment involved conventional cell counting methods. For cell counting, a density of 2 × 10^4^ cells per well was plated into a 6-well plate. These cells were then allowed to incubate for the specified time periods before being counted.

### 2.10. Anchorage-Independent Growth

For the assessment of colony-forming efficiency within a semi-solid medium, a total of 10,000 cells were seeded into a culture medium containing 0.3% agarose. This was placed over a foundational layer of serum-free medium supplemented with 0.5% agarose. Following an incubation period of three weeks, the resulting colonies were quantified. This experimental setup was conducted using 60 mm dishes, and the entire procedure was carried out in triplicate.

### 2.11. In Vivo Xenograft Tumor Assay

A quantity ranging from five to ten million cells was introduced through subcutaneous injection into the flank of immunocompromised mice (strain nu/nu, obtained from Charles River Laboratories, Wilmington, MA, USA). The entire animal study was carried out in strict adherence to the protocol that had been approved by the UCLA Animal Research Committee. To gauge the progression of tumor growth, the volume of the nodules was assessed by measuring three perpendicular axes utilizing micro-scaled calipers. This process enabled the determination of the kinetics involved in the growth of the tumors.

### 2.12. ALDH1 Assay

The determination of ALDH enzymatic activity was carried out using the Aldehyde Dehydrogenase-Based Cell Detection Kit from STEMCELL. A total of 1 × 10^6^ cells were resuspended in a volume of 1 mL of the ALDEFLUOR Assay Buffer. To measure ALDH enzymatic activity in the intact cells, a non-toxic fluorescent substrate called ALDEFLUOR Reagent BODIPYTM (1.25 μL) was introduced.

Directly following the addition of the substrate reagent, 0.5 mL of the cell suspension was transferred into a control tube containing a specific inhibitor for ALDH, diethylaminobenzaldehyde (DEAB). This facilitated the calculation of background fluorescence. The cells were subsequently incubated at a temperature of 35 °C for a duration of 30 min. The acquisition of fluorescence data was conducted using a BD FACScan flow cytometer from BD Biosciences.

### 2.13. Small Interfering RNA (siRNA) Transfection

Orai3 siRNA (sc-76005; Santa Cruz), ID1 siRNA (sc-29356; Santa Cruz, CA, USA), and control siRNA (sc-37007; Santa Cruz, CA, USA) were procured for the study. These siRNAs were introduced into cells using Lipofectamine RNAiMAX from Invitrogen. Specifically, cells numbering 2 × 10^5^ were seeded into 60 mm dishes and subsequently transfected with 10 μg of the respective siRNA. The cultures were collected for analysis one day following the transfection for the purpose of expression and functional assessments.

### 2.14. Mouse Models

To initiate oral tumors, C57BL/6 mice obtained from the Jackson Laboratory were subjected to exposure to 4-Nitroquinoline 1-oxide (4-NQO) obtained from Sigma-Aldrich, St. Louis, MO, USA. This exposure was achieved by diluting 4-NQO in drinking water to attain a final concentration of 30 μg/mL. This exposure protocol spanned 16 weeks, followed by a subsequent six-week period during which the mice consumed normal drinking water. This approach closely followed the methodology outlined in our prior study [35]. Upon the conclusion of the experiment, the tongues of the mice were collected for histological analysis and RNA isolation. All actions involving the use of mice were conducted in strict accordance with the guidelines set forth by the National Institutes of Health and were duly approved by the UCLA Animal Research Committee.

### 2.15. Statistical Analysis

Statistical analyses were conducted using GraphPad Prism 5 software. The data were presented as the mean ± standard deviation (SD). Comparisons between two groups were carried out employing either the parametric Student’s *t*-test or the paired *t*-test, depending on the nature of the data. A significance level of *p* < 0.05 was adopted to determine statistical significance.

## 3. Results

### 3.1. Stepwise Increase in Orai3 Expression during Oral Carcinogenesis

**Figure 1:**
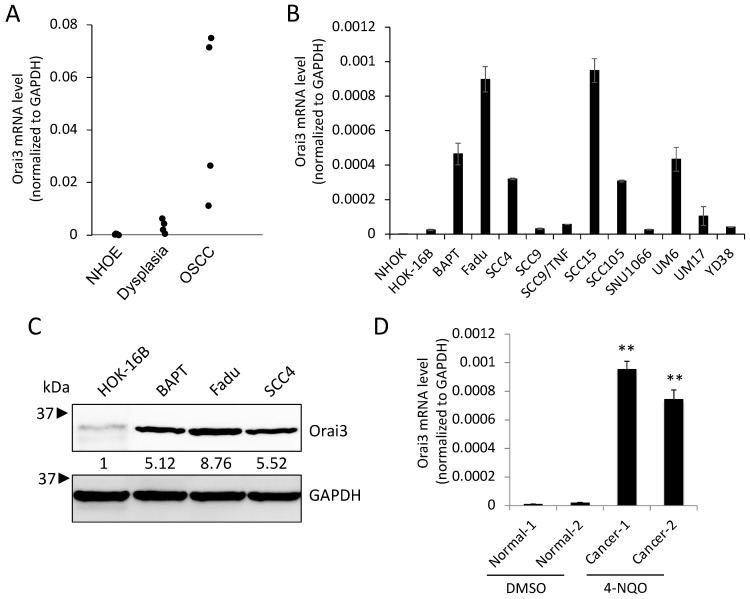
Elevated expression of Orai3 during oral/oropharyngeal carcinogenesis. (**A**) Level of Orai3 mRNA was determined in normal human oral epithelia (NHOE: *n* = 4), oral dysplasia (*n* = 4), and OSCC (*n* = 4) tissues by microdissection followed by qPCR. The levels of Orai3 mRNA were normalized with the expression of GAPDH. Orai3 mRNA values were obtained from three independent experiments. (**B**) Level of Orai3 mRNA was determined in normal human oral keratinocyte (NHOK), non-tumorigenic immortalized oral epithelial cell line (HOK-16B), and 11 OSCC cell lines (BapT, FaDu, SCC4, SCC9, SCC9/TNF, SCC15, SCC105, SNU1066, UM6, UM17B, and YD38) by qPCR. Each qPCR analysis was performed in triplicates. (**C**) Level of Orai3 protein was determined by Western blot. GAPDH was used as a loading control. Densitometric quantification was performed using ImageJ, and the relative band intensity was normalized against GAPDH. Relative numeric protein levels were indicated under the corresponding bands. (**D**) Level of Orai3 was determined in tongues from mice exposed to DMSO or 4-NQO by qPCR. qPCR was performed with total RNAs isolated from tongue tissues obtained from two DMSO treated and two 4-NQO-treated mice. Data are means ± SD of three independent assays. ** *p* < 0.01 compared to Normal-1 and -2.

To investigate the role of Orai3 in oral carcinogenesis, we determined the transcript level of Orai3 in normal human oral epithelia (NHOE), dysplasia, and OSCC tissues by employing laser capture microdissection (LCM), followed by qPCR. The assay revealed a gradual increase in the Orai3 expression during oral carcinogenesis (Figure 1A). The expression of Orai3 in normal human oral keratinocytes (NHOK), immortalized non-tumorigenic oral epithelial cell line (HOK-16B), and OSCC cell lines (BapT, FaDu, SCC4, SCC9, SCC9/TNF, SCC15, SCC105, SNU1066, UM6, UM17B, and YD38) were also evaluated (Figure 1B,C). Orai3 was commonly elevated in the OSCC cells compared to the tested normal and immortalized cells, although with a certain degree of variability among the tested OSCC cell lines (Figure 1B). Consistent with the result from Figure 1A, a gradual increase in Orai3 expression was detected within an in vitro sequential, multistep model of oral carcinogenesis, specifically NHOK→HOK-16B→ BapT (Figure 1B). In this model, NHOK cells were immortalized through high-risk HPV-16 (HOK-16B), and subsequently, the HOK-16B cells underwent transformation into oncogenic cells following exposure to the chemical carcinogen benzo(a)pyrene (BapT) [36]. To further substantiate the significance of the elevated Orai3 level in the context of oral carcinogenesis, we employed a mouse model of tongue cancer induced by carcinogens. As demonstrated in our prior study [35], prolonged exposure of mice to 4-nitroquinoline-1-oxide (4-NQO) resulted in extensive development of oral tumors within the tongue. In contrast, tongues from mice exposed to DMSO (used as the vehicle) displayed comparable histological features to those of normal squamous epithelium. In alignment with our observations in human cancer cases, a significant increase in Orai3 expression was evident in the tongues bearing tumors compared to the tongues with a normal status. This indicates that the heightened expression of Orai3 is linked with chemical-induced oral carcinogenesis (Figure 1D). Collectively, our findings indicate a stepwise increase in Orai3 during oral carcinogenesis, suggesting an important role of Orai3 in the progression of OSCC.

### 3.2. Orai3 Is Enriched in CSC Populations Derived from OSCC

**Figure 2:**
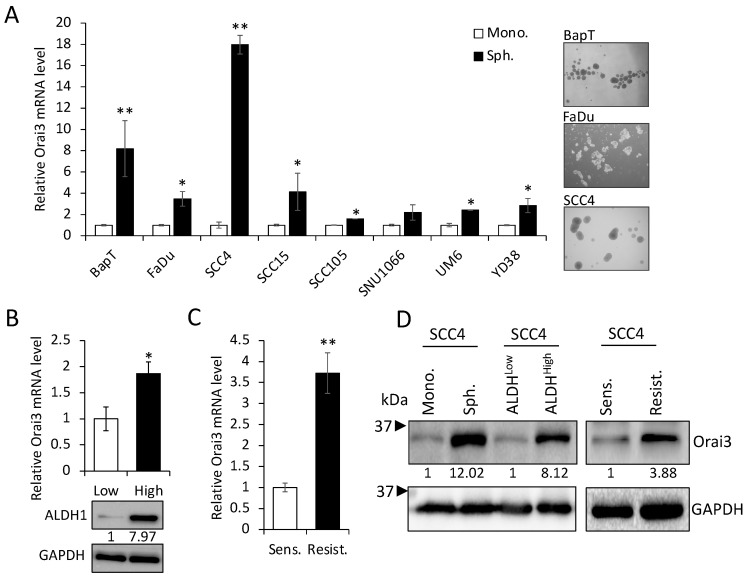
Enrichment of Orai3 in CSC populations. (**A**) Expression of Orai3 was assessed in tumor spheres (Sph.) and their corresponding adherent monolayer cells (Mono.) derived from multiple OSCC cell lines by qPCR. Data are means ± SD of three independent assays. * *p* < 0.05 and ** *p* < 0.01 compared to controls. On the right, a representative image of spheres derived from OSCC cell lines was displayed. (**B**) ALDH1^HIGH^ (High) and ALDH1^low^ (Low) cell populations were sorted from SCC4 cells by flow cytometry, and their Orai3 expression level was assessed by qPCR (upper) and Western blot (lower). Relative numeric protein levels were indicated under the corresponding bands. * *p* < 0.05. (**C**) Expression of Orai3 was assessed in cisplatin-sensitive (Sens) and cisplatin-resistant (Resist) SCC4 cells by qPCR. Orai3 levels were obtained from three independent qPCRs. ** *p* < 0.01. (**D**) Expression of Orai3 was assessed in CSC populations and their corresponding non-CSC populations by Western blot. Relative numeric protein levels were indicated under the corresponding bands.

Emerging evidence indicates that CSCs play a key role in cancer progression [37]. Thus, to explore the importance of Orai3 in CSCs, we first compared the levels of Orai3 in various CSC populations with those in their corresponding non-CSC populations derived from OSCC cells. Tumor spheres originate from self-renewing cells and exhibit an augmented CSC phenotype along with an elevated expression of stemness transcription factors (Nanog, Oct4, KLF4, Lin28, and Sox2), in contrast to their corresponding monolayer adherent cell counterparts. This observation underscores their status as a cell population enriched with CSC attributes [14]. Orai3 was highly enriched in tumor spheres compared with their corresponding monolayer adherent cells derived from multiple OSCC cell lines (Figure 2A). The activity of aldehyde dehydrogenase 1 (ALDH1) is a key CSC marker for head and neck cancer, including OSCC [2]. ALDH1^High^ cancer cells display enhanced CSC properties compared to ALDH1^Low^ cells [2]. In our study, ALDH1^High^ OSCC cells expressed higher levels of Orai3 transcript than ALDH1^Low^ OSCC cells sorted from SCC4 (Figure 2B). The population of CSCs has the potential to become more concentrated following the administration of chemotherapeutic drugs [2]. Thus, we also measured the Orai3 expression in cisplatin-resistant SCC4 cells that were isolated from SCC4 treated with 25 μM cisplatin for 2 days [38]. Orai3 mRNA was significantly elevated in the cisplatin-resistant cells compared to their sensitive control cells (Figure 2C). Moreover, Orai3 protein was also greatly increased in the tested CSC populations compared to their corresponding non-CSC populations (Figure 2D). Our findings propose that elevated Orai3 expression might be indispensable for sustaining CSCs in OSCC.

### 3.3. Elevated Orai3 Supports CSC Phenotype

**Figure 3:**
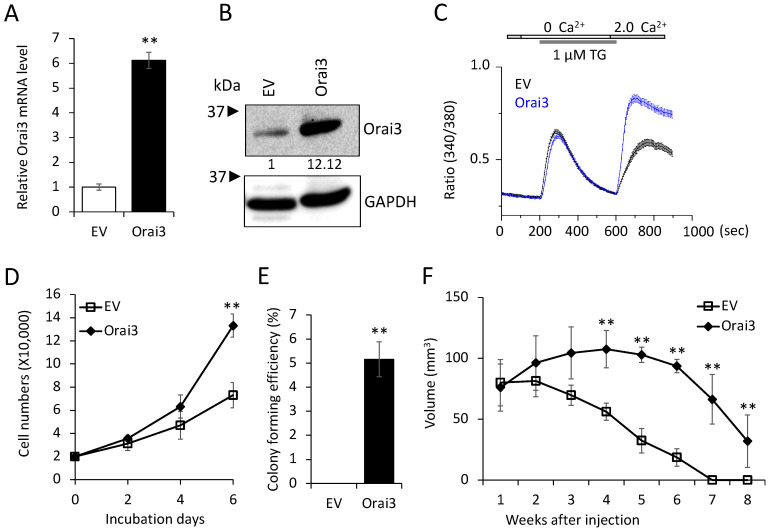
Ectopic Orai13 expression induces malignant growth properties in non-tumorigenic immortalized oral epithelial cells. Orai3 was overexpressed in non-tumorigenic immortalized oral epithelial cells, HOK-16B, by infecting with retroviral vector expressing Orai3, and its overexpression was confirmed by (**A**) qPCR and (**B**) Western blot analysis. Relative numeric protein levels were indicated under the corresponding bands. ** *p* < 0.01. (**C**) Intracellular Ca^2+^ imaging assay was performed to confirm the activation of Orai3 function. Intracellular Ca^2+^ stores were depleted with 1 μM TG in the absence of extracellular Ca^2+^, followed by re-addition of 2 mM Ca^2+^. Ca^2+^ influx was analyzed by single-cell video imaging of Fura2-labeled, GFP+ cells. More than 30 GFP+ cells were analyzed in each experiment. (**D**) Effect of Orai3 on cell proliferation of HOK-16B was determined by cell counting. Data are means ± SD of triplicate experiments. ** *p* < 0.01. (**E**) Effect of Orai3 on anchorage independent growth of HOK-16B was determined by soft agar assay. Data are means ± SD of triplicate experiments. ** *p* < 0.01. (**F**). Effect of Orai3 on in vivo tumorigenicity of non-tumorigenic immortalized oral epithelial cells was determined by xenograft tumor assay. HOK-16B/EV and HOK-16B/Orai3 were injected subcutaneously into 5 nude mice. Tumor sizes were measured for 8 weeks. ** *p* < 0.01.

Having established that increased Orai3 is necessary for CSCs, we next examined whether ectopic Orai3 expression confers CSC phenotype on oral epithelial cells. We overexpressed Orai3 in non-tumorigenic immortalized oral epithelial cells, HOK-16B, using the lentiviral vector expressing Orai3 or an empty vector (EV) as a control (Figure 3A,B). The elevation of Ca^2+^ influx by Orai3 was also confirmed by performing a Ca^2+^ imaging assay (Figure 3C). Then, we examined the effect of Orai3 on cell proliferation and found that Orai3 overexpression led to a robust increase in the proliferation capacity of HOK-16B in vitro (Figure 3D). Moreover, Orai3 imparted the capacity for anchorage-independent growth to HOK-16 cells (Figure 3E). This attribute has been associated with heightened tumor cell aggressiveness in vivo, encompassing traits like tumorigenicity [39]. To further examine the effect of Orai3 on tumor growth in vivo, we injected the cells into nude mice and observed tumor formation (Figure 3F). In the 1st week following injection, both HOK-16/EV and HOK-16B/Orai3 cells generated nodules of comparable sizes. Nodules formed by HOK-16B/EV cells began regressing in the 2nd week after injection and had fully regressed by the 8th week. Conversely, HOK-16B/Orai3 cells continued nodule growth into the 2nd week after injection, reaching their peak dimensions by the 4th week. Subsequently, these nodules underwent regression, resulting in the presence of necrotic tissue by the 8th week. Our findings indicate that ectopic expression of Orai3 in the non-tumorigenic immortalized oral epithelial cells resulted in an increase in the cell proliferation and the acquisition of malignant growth properties.

**Figure 4:**
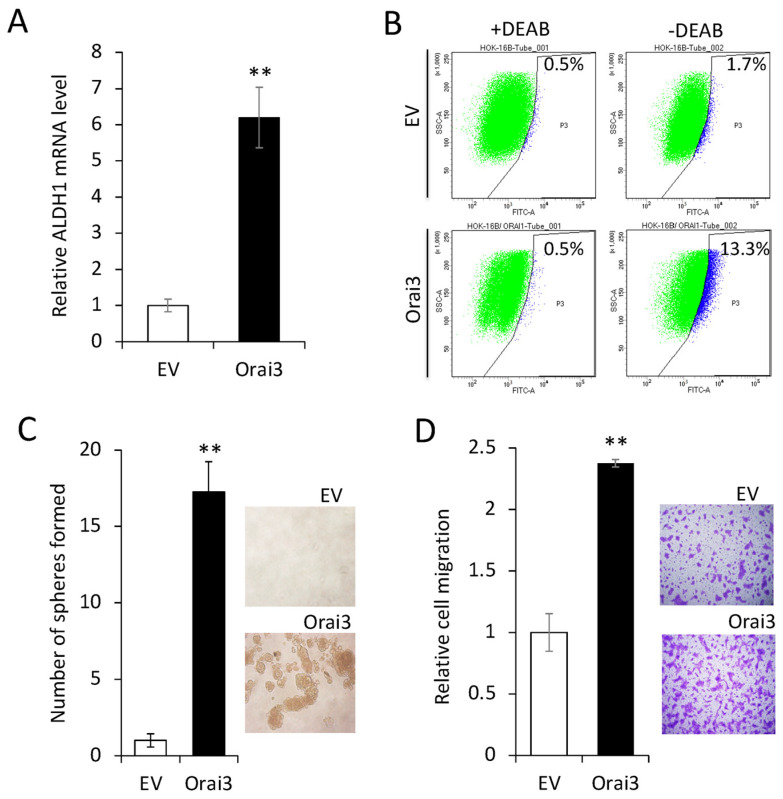
Ectopic Orai3 expression enhances CSC phenotype in non-tumorigenic immortalized oral epithelial cells. (**A**) Effect of Orai3 on ALDH1 expression in HOK−16B was determined by qPCR. Data are means ± SD of three independent assays. ** *p* < 0.01. (**B**) Effect of Orai3 on ALDH1 activity in HOK−16B was determined by Aldefluor assay. Cells were labeled with Aldefluor combined with or without the ALDH1 inhibitor DEAB and analyzed by flow cytometry. The gate for ALDH1 + cells was determined in relation to the DEAB control (+DEAB) and shows the brightly fluorescent ALDH1 population versus the side scatter, a population that is absent/decreased in the presence of DEAB. The number shown in each panel reflects the percentage of ALDH1+ cells in each cell type. (**C**) Effect of Orai3 on self-renewal capacity of HOK-16B was determined by tumor sphere formation assay. Representative image of tumor spheres formed by HOK−16B/EV and HOK−16B/Orai3 are shown on the right. Bar indicates 100 μm. ** *p* < 0.01. (**D**) Effect of Orai3 on migration ability in HOK−16B was determined by transwell migration assay. Data were obtained from three independent assays. ** *p* < 0.01.

Next, we investigated the effect of Orai3 on the CSC phenotype in the immortalized cells. The ectopic Orai3 expression significantly increased both the ALDH1 mRNA by 6-fold (Figure 4A) and the ALDH1^High^ cell population by 7.8-fold (1.7% vs. 13.3%, Figure 4B) in HOK-16B, indicating that Orai3 increases the CSC population. Orai3 expression induced tumor sphere formation ability, indicating the acquisition of self-renewal capacity by Orai3 (Figure 4C). A transwell migration assay (Figure 4D) demonstrated that HOK-16B/Orai3 migrated significantly faster than HOK-16B/EV. These findings indicate that ectopic Orai3 expression is sufficient to confer CSC phenotype on the immortalized oral epithelial cells.

**Figure 5:**
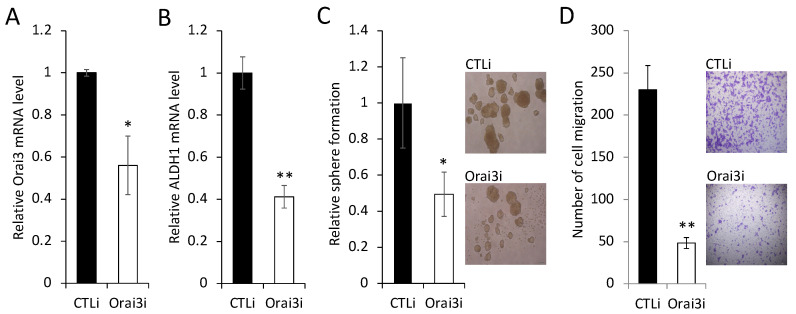
Inhibition of Orai3 suppresses CSC phenotype in OSCC cells. (**A**) Endogenous Orai3 was knocked down in SCC4 using siRNA against Orai3 (Orai3i). The cells transfected with control siRNA (CTLi) were included for comparison. Knockdown of Orai3 was confirmed by qPCR. Data are means ± SD of three independent assays. * *p* < 0.05. (**B**) The effect of Orai3 knockdown on ALDH1 was determined by qPCR. ** *p* < 0.01. (**C**) The effect of Orai3 knockdown on self-renewal capacity was determined by tumor sphere formation assay. Data are means ± SD of three independent experiments. * *p* < 0.05. Bar indicates 100 μm. (**D**) The effect of Orai3 knockdown on migration ability was determined by transwell migration assay. Data are means ± SD of three independent experiments. ** *p* < 0.01. Bar indicates 100 μm.

To further confirm the importance of elevated Orai3 expression for the maintenance of the CSC phenotype, we knocked down endogenous Orai3 in OSCC cells by siRNA (Figure 5A). Knockdown of Orai3 suppressed the expression of ALDH1 mRNA by 60% (Figure 5B), self-renewal capacity by 50% (Figure 5C), and migration ability by 95% (Figure 5D) of the OSCC cells. These findings clearly indicate that Orai3 is required for the maintenance of the CSC phenotype in OSCC. Collectively, our data suggest that Orai3 promotes OSCC progression by increasing cancer stemness.

### 3.4. ID1 Is Required for Orai3-Induced CSC Phenotype

**Figure 6:**
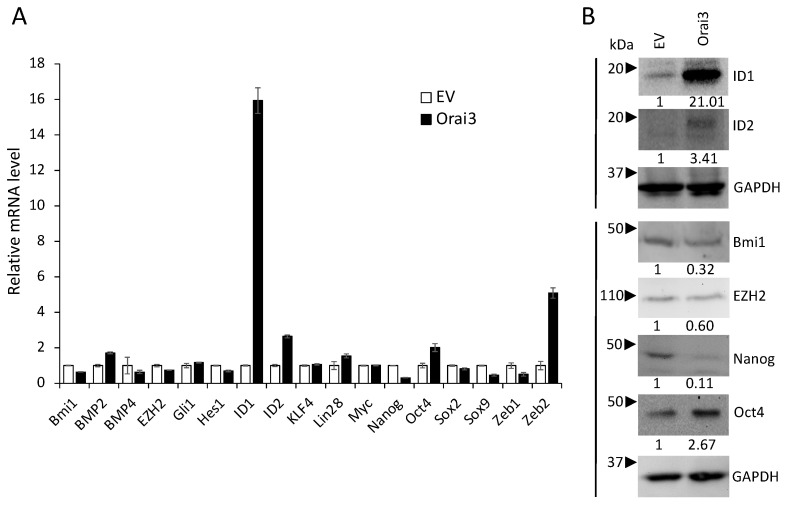
Orai3 increases ID1 expression. Effect of Orai3 on CSC-related genes was determined by (**A**) qPCR and (**B**) Western bot analysis. Their levels in HOK-16B/Orai3 were plotted as fold change against those in HOK-16B/EV. qPCR data are means ± SD of three independent assays. Relative numeric protein levels were indicated under the corresponding bands.

To understand the mechanism by which Orai3 regulates the CSC phenotype, we initially examined the effect of Orai3 on the expression of 17 pivotal regulators associated with CSCs (Figure 6A). Among the tested CSC regulators, inhibitor of DNA binding 1 (ID1), a stemness transcription factor, was most upregulated by Orai3 in HOK-16B (Figure 6A,B). Conversely, the silencing of Orai3 resulted in a significant decrease in ID1 expression (Figure S1). Since ID1 is considered a master regulator of CSCs [40] and plays an oncogenic role in OSCC [41,42,43,44], our findings suggest that ID1 may play an important role in Orai3-mediated CSC regulation.

**Figure 7:**
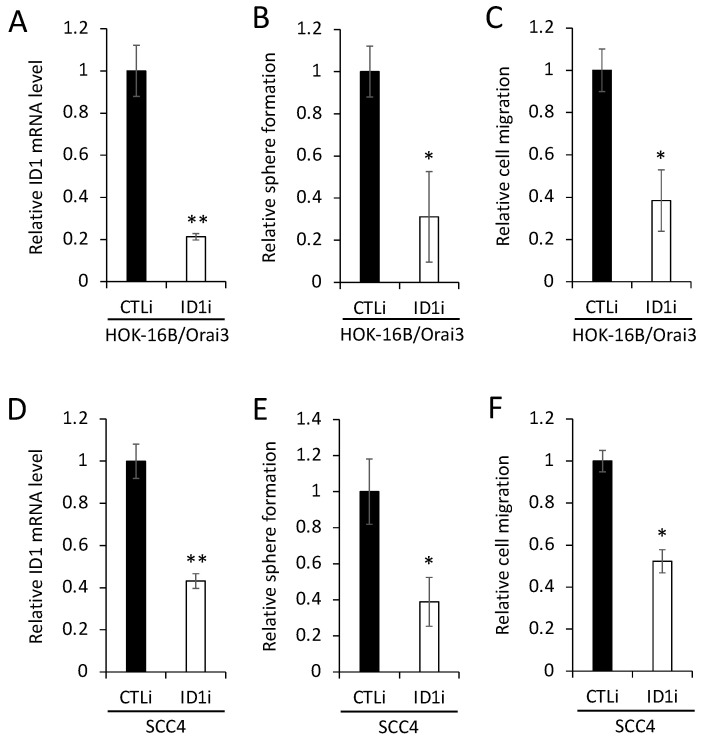
ID1 is required for Orai3-induced CSC phenotype. (**A**) ID1 was knocked down in HOK-16B/Orai3 using siRNA against ID1 (ID1i). The cells transfected with control siRNA (CTLi) were included for comparison. Knockdown of ID1 was confirmed by qPCR. The assay was performed in triplicates. (**B**) The effect of ID1 knockdown on self-renewal capacity of HOK-16B/Orai3 was determined by tumor sphere formation assay. The assay was performed in triplicates. (**C**) The effect of ID1 knockdown on migration ability in HOK-16B/Orai3 was determined by transwell migration assay. The assay was performed in triplicates. (**D**) Endogenous ID1 was knocked down SCC4 using siRNA against ID1 (ID1i). The cells transfected with control siRNA (CTLi) were included for comparison. Knockdown of ID1 was confirmed by qPCR. (**E**) The effect of ID1 knockdown on self-renewal capacity of SCC4 was determined by tumor sphere formation assay. (**F**) The effect of ID1 knockdown on migration ability of SCC4 was determined by transwell migration assay. * *p* < 0.05, ** *p* < 0.01.

To evaluate the functional role of ID1 in the Orai3-induced CSC phenotype, we knocked down ID1 in HOK-16B/Orai3 by siRNA (Figure 7A). The knockdown of ID1 reduced self-renewal by 75% (Figure 7B) and migration by 60% (Figure 7C) in the cells with ectopic Orai3 overexpression. We also demonstrated that the knockdown of endogenous ID1 in OSCC (Figure 7D) suppressed self-renewal by 60% (Figure 7E) and migration ability by 50% (Figure 7F). Taken together, our data indicate that Orai3 promotes the CSC phenotype through the elevation of ID1, suggesting a novel CSC regulatory mechanism by the Orai3/ID1 axis.

### 3.5. Orai3 and ID1 Are Highly Expressed in CSC-Enriched Populations, and Their Expression Levels Are Positively Correlated in OSCC Cells

**Figure 8:**
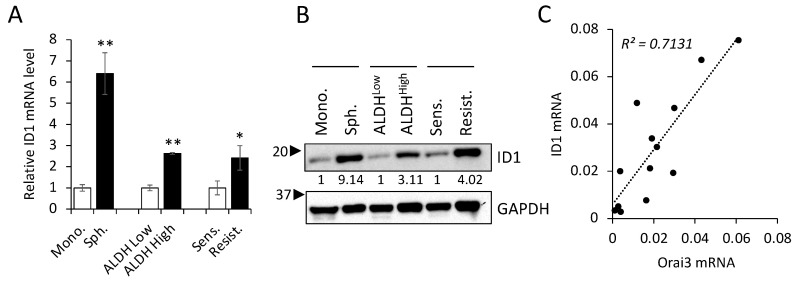
Orai3/ID1 axis is enriched in CSC populations, and their mRNA levels are positively correlated in OSCC cells. Expression of ID1 is measured in CSC populations and their corresponding non-CSC populations by (**A**) qPCR and (**B**) Western blot. qPCR data are means ± SD of three independent experiments. Relative numeric protein levels were indicated under the corresponding bands. * *p* < 0.05, ** *p* < 0.01 (**C**) Correlation analysis of Orai3 and ID1 mRNA was determined based on their expression levels in 13 human OSCC cell lines by qPCR.

To gain deeper insights into the significance of the Orai3/ID1 axis in CSCs, we quantified the levels of ID1 in various CSC populations compared with those in their corresponding non-CSC populations (Figure 8A,B). Similar to the enrichment of Orai3 in CSCs (Figure 2), ID1 exhibited elevated expression levels in tumor spheres in comparison to their corresponding monolayer adherent cells. ALDH1^High^ OSCC cells expressed higher ID1 than ALDH1^Low^ OSCC cells sorted from SCC4. Moreover, the level of ID1 was much greater in cisplatin-resistant than cisplatin-sensitive SCC4 cells. These findings indicate that the Orai3/ID1 axis is elevated in CSC-enriched OSCC populations. Additionally, in order to ascertain whether there exists a correlation between the expression of Orai3 and ID1 in OSCC, we conducted a comparison of Orai3 and ID1 levels across 13 human OSCC cell lines (Figure 8C). Our findings demonstrated a positive correlation between the transcript levels of Orai3 and ID1 within the context of OSCC.

## 4. Discussion

The CSC hypothesis is a well-studied theory to explain the initiation and progression of tumors [2]. CSCs represent a minority within the diverse population of tumor cells, and they possess distinctive attributes that allow them to sustain tumor aggressiveness. These characteristics include the capacity for self-renewal, the ability to migrate, and resistance to therapeutic drugs [2]. CSCs have been successfully isolated from various solid tumors, including OSCC, a prevalent malignancy affecting the head and neck region. In our previous work, we have elucidated numerous molecular factors that govern the stem-like properties of OSCC and proposed their potential utilization in therapeutic approaches [14,45,46,47]. Owing to the heterogeneity of CSCs [48], unveiling novel molecular regulators for cancer stemness is of paramount importance for developing a new generation of effective therapies for OSCC. To the best of our knowledge, this is the first study to report that Orai3/ID1 signaling is a novel molecular axis for governing the stemness of OSCC. The expression of Orai3 is elevated in a stepwise manner during OSCC carcinogenesis and further enriched in CSC populations compared to that in non-CSC populations. Ectopic expression of Orai3 in immortalized oral epithelial cells induces malignant growth as well as a CSC phenotype. Inhibition of Orai3 suppresses cancer stemness of OSCC. Furthermore, our data indicate that Orai3 enhances the CSC phenotype through the elevation of ID1, suggesting the vital role of the Orai3/ID1 axis in OSCC CSCs.

The emerging pathophysiological role of Orai3 has been reported in various solid cancers, such as breast, prostate, lung, colorectal, and pancreatic cancer [49]. Elevated expression of Orai3 has been observed in various human cancers, including breast, prostate, and pancreatic cancer [49]. However, there is no report on the role of Orai3 in oral/oropharyngeal carcinogenesis. Our study showed that Orai3 was highly expressed in OSCC compared to precancerous and normal tissues and culture cells. Furthermore, precancerous oral epithelial cells expressed higher Orai3 compared to normal oral epithelial cells. These findings indicated a stepwise increase in the Orai3 expression during oral/oropharyngeal carcinogenesis. Moreover, Orai3 expression was greatly elevated in a carcinogen-induced tongue cancer mouse model. Our findings indicate that Orai3 plays a vital role in the progression of OSCC in vivo.

Our study clearly demonstrated that Orai3 is required for the maintenance of malignant growth and the stemness properties of OSCC. Ectopic Orai3 expression in immortalized oral epithelial cells induced not only Ca^2+^ influx but also malignant growth properties, including anchorage-independent growth ability and self-renewal capacity. Anchorage-independent growth ability is a well-known characteristic of cancer, which is linked to in vivo tumorigenicity [39]. Self-renewal capacity is considered as the key characteristic by which CSCs regenerate themselves, suggesting the driving force of tumorigenesis. Indeed, Orai3 increased the cell proliferation capacity in vivo; however, Orai3 failed to endow the cells with full tumor-forming ability in vivo. Furthermore, Orai3 increased the CSC populations and phenotype. Ectopic Orai3 expression markedly increased the ALDH1^High^ CSC population in the non-tumorigenic oral epithelial cells. The ALDH1^High^ cancer cells displayed higher self-renewal, migration, and tumorigenic potential than the ALDH1^low^ cells [50,51,52]. Orai3 also markedly increased the motility of the non-tumorigenic cells. Conversely, inhibition of endogenous Orai3 in OSCC resulted in reduced CSC properties, such as ALDH1 activity, self-renewal capacity, and migration ability. Our finding aligns with prior studies underscoring the significance of Orai3 in cell migration. Specifically, in invasive breast cancer cell lines, the reduction of Orai3 through knockdown led to a decline in cell migration, while the augmentation of Orai3 expression resulted in an enhancement of cellular motility [53]. Nevertheless, the precise mechanism through which Orai3 augments the migration of oral epithelial cells remains elusive. Consequently, a comprehensive exploration of the impact of Orai3 on epithelial-to-mesenchymal transition (EMT) and the expression of genes associated with metastasis is imperative to unravel these effects [54]. We conclude that Orai3 increases not only the number of CSCs but also the CSC properties. Hence, our working hypothesis postulates that Orai3 plays a role in propelling the malignant advancement of OSCC through the augmentation of the CSC phenotype. Collectively, our findings suggest that Orai3 holds the potential to serve as a promising therapeutic target for OSCC.

Orai3 regulates cancer growth and migration through multiple pathways [49]. Our study demonstrated that Orai3 enhanced the CSC phenotype via the upregulation of ID1 (inhibitor of DNA-binding 1), a stemness transcription factor. ID proteins lack a DNA binding domain and function as dominant negative regulators of basic helix-loop-helix (bHLH) transcription factors through heterodimerization with other bHLH factors [55]. Among four ID family members (ID1-4), ID1 is the most extensively studied, and its role in cancer is generally considered as a tumor promoter [56]. ID1 is overexpressed in many types of malignancies, such as breast, lung, prostate, cervical, colorectal, liver, and brain cancer. ID1 enhances tumor progression, aggressiveness, and metastasis which are responsible for mortality in cancer patients. Conversely, ID1 inhibition results in a decrease in tumor growth and metastasis [57]. ID1 elicits its tumor-promoting effects by regulating various target genes involved in cancer development [56]. For instance, ID1 promoted lung cancer growth by activating CDK4/cyclin D1 [58] and breast cancer metastasis through S100A9 regulation [59]. Interestingly, a potential role of ID1 in the regulation of cancer stemness has been demonstrated. ID1 promoted the stemness phenotype through the activation of the WNT/SHH signaling pathway in brain and colorectal cancer [60,61]. Moreover, ID1 induced breast carcinogenesis by increasing stem cell activities in transgenic mice [62]. These findings suggest that ID1 may mediate its tumor-promoting effects by regulating cancer stemness. Genetic inhibition of ID1 in OSCC cells suppressed cancer stemness features, including self-renewal and migration. Moreover, the ID1 inhibition in HOK-16B/Orai3 led to suppression of the CSC phenotype, indicating the functional role of increased ID1 in the Orai3-induced CSC phenotype. This suggests that the Orai3/ID1 axis is a novel regulatory signaling mechanism for maintaining cancer stemness in OSCC. As secondary evidence supporting this, we found that both Orai3 and ID1 are enriched in CSC populations, and their expression is positively correlated. Consequently, we hypothesize that Orai3 contributes to cancer stemness through the elevation of ID1 expression. Nevertheless, the intricate mechanisms underpinning how Orai3 modulates ID1 expression remain to be fully comprehended.

Orai channels induce Ca^2+^ entry, and increased intracellular Ca^2+^ activates downstream responses, including the NFAT signaling pathway [63]. The activation of NFAT signaling holds a crucial role in the process of tumorigenesis, as it oversees the regulation of a multitude of target genes that participate in the development of cancer [64]. Indeed, NFAT constitutes a transcription factor family comprised of four distinct members, namely NFATc1, NFATc2, NFATc3, and NFATc4 [65]. To test if NFAT signaling is activated by Orai3, we examined the expressions of NFAT in HOK-16B/Orai3 cells. Interestingly, among the four members, only NFATc1 expression was upregulated in the cells, suggesting that increased intracellular Ca^2+^ by Orai3 activates NFATc1, implying a novel Orai3-NFATc1-ID1 circuit. However, the role of NFATc1 on ID1 regulation has not been documented. Therefore, further investigation is necessary to examine the detailed regulatory mechanism of the Orai3-NFATc1-ID1 circuit and its role in the regulation of CSCs. Moreover, the Ca^2+^-independent mechanism of Orai3 was reported in cancer [66], which adds an additional layer of interest for the future study of Orai3.

In conclusion, the Orai3 Ca^2+^ channel is a novel molecular regulator of the malignant and stemness phenotype of OSCC. Orai3 promotes the CSC phenotype via ID1 upregulation. The Orai3/ID1 axis is augmented in CSC populations of OSCC, suggesting that the Orai3/ID1 axis could be an important therapeutic target in OSCC. Given the susceptibility of both Orai3 and ID1 to inhibition by small molecular agents [67,68], targeting the Oria3/ID1 axis could be a selective therapeutic modality against OSCC CSCs.

## Data Availability

All data are available in the text and supplementary materials.

## Supplementary Materials

The following supporting information can be downloaded at: https://www.mdpi.com/article/10.3390/cells12182225/s1, Figure S1: Effect of Orai3 knockdown on CSC-related genes determined by qPCR. Orai3 was knocked down in HOK-16B/Orai3 using siRNA against Orai3 (Orai3i). The cells transfected with control siRNA (CTLi) were included for comparison. * *p* < 0.05 and ** *p* < 0.01 compared to the CTLi transfected control.

## Abbreviations

Ca^2+^: calcium; OSCC, Oral/oropharyngeal squamous cell carcinomas; CSCs, Cancer stem-like cells; ID1, inhibitor of DNA binding 1; NFAT, nuclear factor of activated T cells; NHOK, normal human oral keratinocytes; NHOE, Normal human oral epithelia; LCM, laser-captured microdissection; ALDH1, Aldehyde dehydrogenase 1; siRNA, small interfering RNA.
