# Supplementary figures and images for: Orai3 Calcium Channel Contributes to Oral/Oropharyngeal Cancer Stemness through the Elevation of ID1 Expression
Source: Cells. 2023 Sep 7;12(18):2225. doi: 10.3390/cells12182225 (PMC10527097; doi:10.3390/cells12182225)

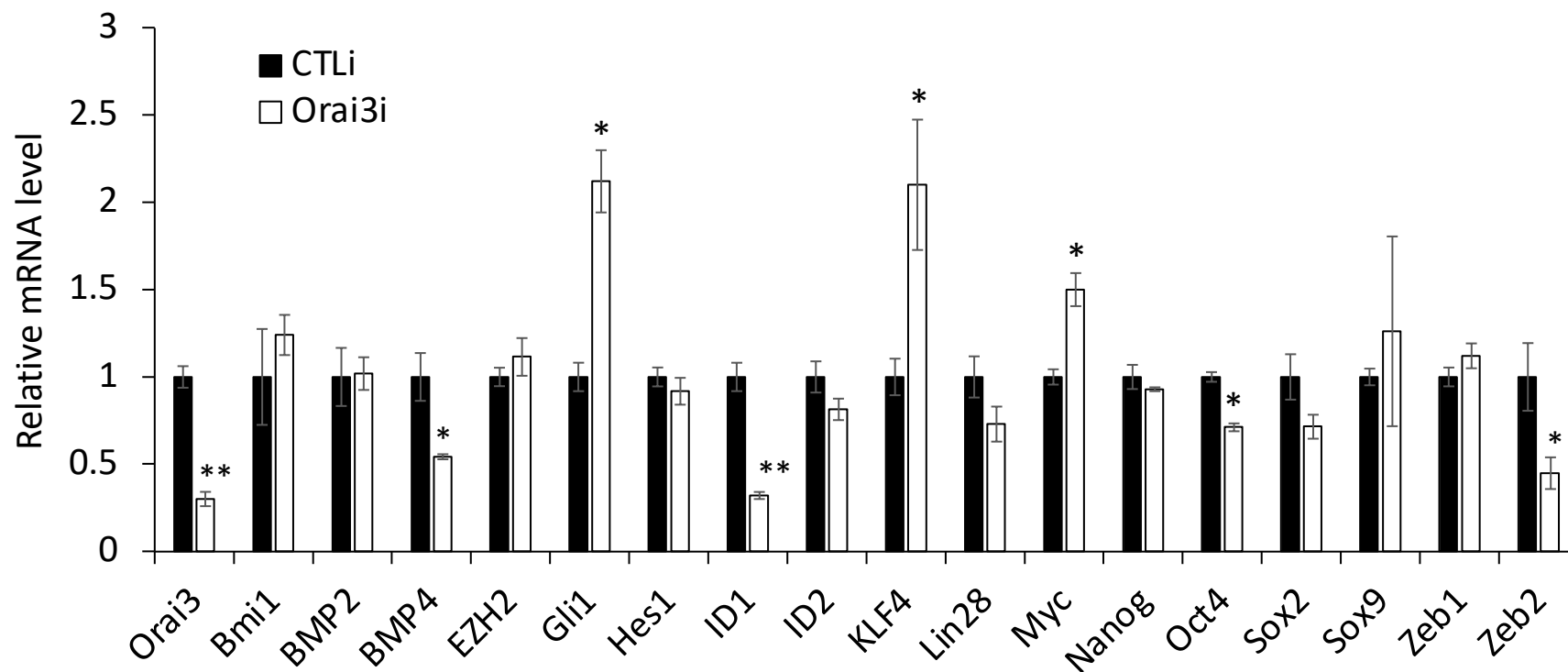

Supplement: Supplementary file 1 [file cells-12-02225-s001.zip › cells-2555117-supplementary.pdf]
